# Design and experiment of impeller seed guide device for rice internal suction hole direct seeding device

**DOI:** 10.1038/s41598-024-64002-x

**Published:** 2024-06-10

**Authors:** Yansong Shang, Bo Zhou, Jitao Yang, Shun Zhang

**Affiliations:** 1 School of Engineering, Anhui Agricultural University, Hefei, 230036 Anhui China; 2 Engineering Laboratory of Intelligent Agricultural Machinery Equipment, Hefei, 230036 Anhui China

**Keywords:** Rice, Internal suction type, Seed metering device, Precision hole-direct seeding, Seed guiding device, Impeller, Engineering, Mechanical engineering

## Abstract

Multi-grain hole-forming sowing and uniform hole spacing are important agronomic requirements for precise hole-direct seeding of rice.This paper designs a kind of impeller type seed guiding device. The main structural parameters of the impeller seed guide device were analyzed by constructing the kinematics model of the rice seed in the impeller seed guide process. The experiment analysis were carried out with the long-grain rice variety Chuangliangyou 4418 as the seeding object. The optimal structural parameter combination of seeding-guiding device was determined as inner impeller radius 56 mm, blade offset angle 11° and seeding angle 36°. On this basis, the seeding performance test of different seed guiding devices of internal suction seed-metering device was carried out by using rice seeds with different external dimensions. The test results show that the impeller has better cavitation and hole spacing uniformity than the seed guide tube. The average hole diameter is not higher than 21.7 mm, the qualified rate of hole diameter is not lower than 96.1%, and the coefficient of variation of hole spacing is not higher than 10.1%. Compared with the seed guide tube, which is increased by 32%, 16% and 34% respectively, and the average hole distance is about 200 mm in theory.

## Introduction

Rice production is an important agricultural production activity to ensure global human food rations^1–3^. It is one of the most important strategic crops around the world^4,5^. Compared with the traditional rice transplanting planting technology, rice direct seeding planting technology has no seedling, seedling raising, seedling transportation, transplanting and other links, which has significant advantages of labor saving, cost saving and efficiency increasing^6–8^. In recent years, direct seeding planting technology and equipment have been widely developed and applied in large areas. The mechanized precision hill direct seeding technology, accurately spacing rice seeds in the field, is widely adopted due to its compatibility with rice cultivation and agronomy^9^. It is of great significance to realize the simplification, mechanization and scale of rice production^10,11^.

The core device of precision hill-drop direct seeding technology is seed-metering device. The seed guiding component is an important part of the seed-metering device, which is used to guide the seeds discharged from the seed-metering device to the seed bed. The existing seed guiding components generally use plastic hoses to adapt to the rise and fall of the seeder opener with the fluctuation of the surface and the adjustment of the planting row spacing. When the seeds are guided in the tube, the random collision between the seeds and the tube wall and the seeds, as well as the random vibration of the seeder, make the delivery time of the seeds in the same hole inconsistent, resulting in poor uniformity of seeding and hole spacing.

In response to issues with the seed guide tube, multiple scholars have proposed various seed guide schemes. Chen et al. developed a belt seed guiding device and determined its primary structural parameters^12,13^. However, there is a gap between the clapboard and the shell on the seed guide belt. For the grains with a small diameter, such as rice seeds and rapeseed, it is easy to be clipped into the gap during the grain guide process, resulting in extrusion friction between the grains and the shell of the seed guide device. Therefore, this delivery method is suitable for grains with large diameter and difficult to be clamped into the gap, such as corn^14^. High-speed grain transportation using positive pressure airflow pipes is widely used in corn, wheat, sesame and other crops, but it is applied to fixed-distance multi-grain hole sowing, and spindle-shaped rice seeds still have great challenges. Its feasibility remains to be verified. In addition, the transportation method also requires additional power consumption^10,15–17^. This paper introduces the design of an impeller seed guide device based on the low energy consumption and stable suction of the inner suction seed-metering device. The suitable key structural parameters of the seed guiding device were determined through seeding performance tests, aiming to enhance the seeding effect of the rice internal suction hill seeding device.

## Materials and methods

### Rice seeds

In order to adapt to the precision hole direct seeding of hybrid rice varieties with different shapes and sizes, three typical size varieties of long-grain type Chuangliangyou 4418, medium-grain type Zhongnong 2008 and short-grain type Yangguang 800 widely planted in the middle and lower reaches of the Yangtze River in China were used as the seeding objects.

### The structure and working principle of impeller guide parts

**Figure 1:**
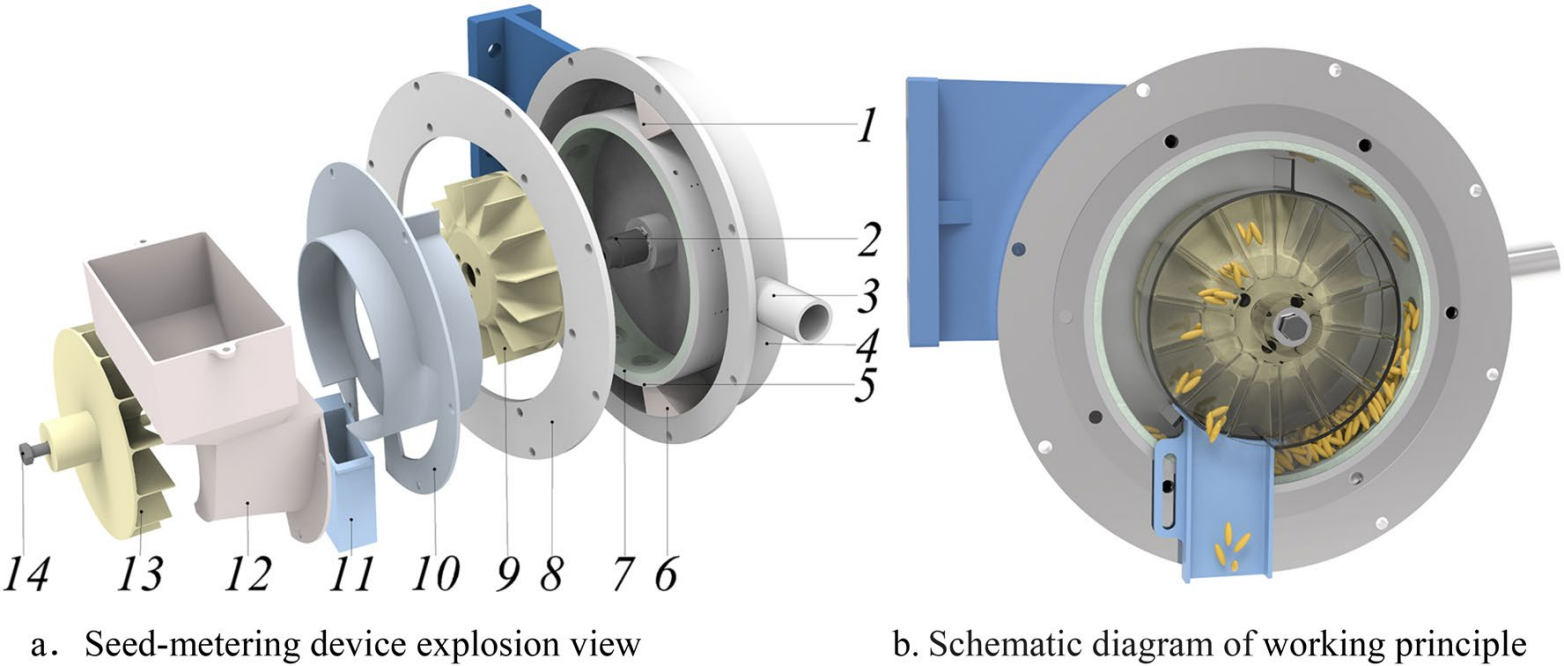
Schematic diagram of seed-metering device. Note: 1. Upper air spacer 2. Sowing shaft 3. Air outlet 4. Shell 5. Seed-absorbed cylinder 6. Lower air spacer 7. Socket ring 8. Air chamber sealing plate 9. Internal impeller 10. Cover plate 11. Protective pipe 12. Seed box 13. External impeller 14. Bolt.

The impeller guide parts are mainly composed of, internal impeller, external impeller, cover plate and protective pipe, as shown in Fig. 1. According to the structural characteristics of the suction seed in the seed-metering device, the impeller guide parts adopt the embedded design idea. The internal and external impellers are positioned circumferentially by the positioning pin, so that the blades of internal and external the two impellers are flush, and are fixed to the drive shaft by key connection and fastening screws. A compartment is formed between two adjacent blades of the internal and external impellers. The number of compartments is same as the number of sockets on the Seed-absorbed cylinder, and is evenly distributed on the circumference of the impeller. Due to the complex structure of the inner and outer impellers and the cover plate, in order to facilitate processing, 3D printing technology is used, and the material is acrylonitrile-butadine-styrene (ABS).

When the seed-metering device works, the seed-absorbed cylinder, the internal impeller, the external impeller and the sowing shaft rotate synchronously. The rice seeds are stably adsorbed by the suction hole and then separated from the seed population. The rice seeds that are not stably adsorbed by the suction hole fall back to the seed population under the action of their self-weight. The adsorbed rice seeds moved to the top with the seed-absorbed cylinder. At this time, the negative pressure of the suction hole was blocked by the upper air spacer. The rice seeds were thrown out from the sockets and fell into the internal impeller compartment, and then guided to the external impeller by the internal impeller slope. With the rotation of the external impeller, when the blade turned the horizontal position and tilted downward to a certain angle, multiple rice seeds in the same compartment began to slide along the blade, and gathered near the cover plate and the blade. Finally, the seed was thrown out through the protective pipe at the outlet to complete the seed guiding process. Among them, the protective pipe protects the throwing trajectory from external factors such as stones and airflow, and does not contact with rice seeds (Supplementary file 1).

### Key structure design of impeller guide parts

#### Internal impeller

**Figure 2:**
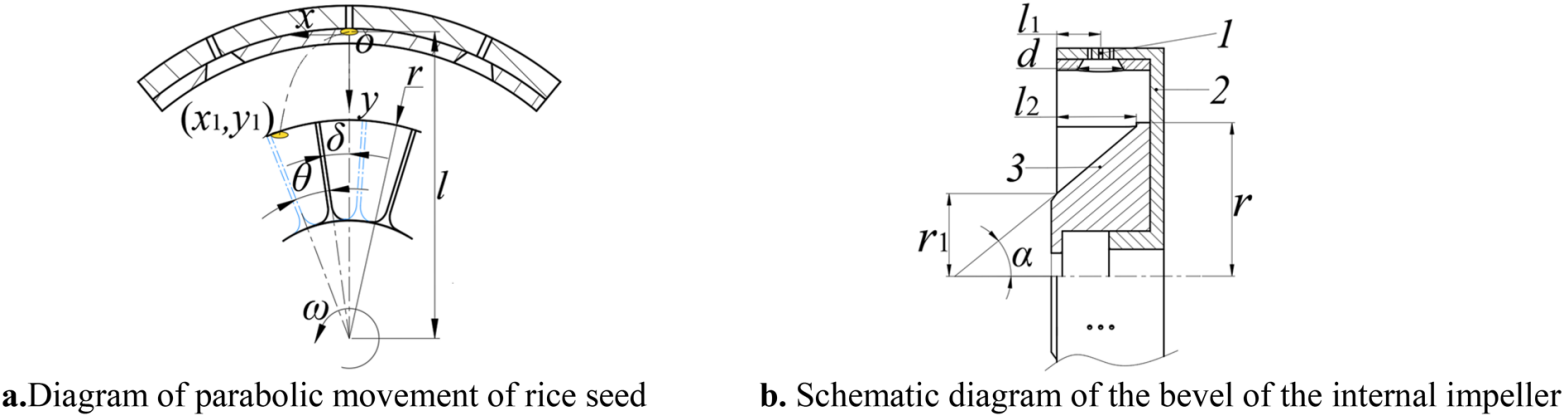
Two perspectives of the inner impeller. Note: 1. Socket ring 2. Seed- absorbed cylinder 3. Internal impeller.

The upper air spacer is positioned on top of the lead hammer in the seed-absorbed cylinder. When the adsorbed rice seed reaches the upper air spacer, the suction negative pressure is blocked, causing the rice seed to be ejected from the cylinder with a flat throwing motion. Taking the centroid of the moment when the rice seed is thrown as the origin, the space rectangular coordinate system is made, as shown in Fig. 2. The equation of rice seed motion trajectory is as follows:

1 $$ \left\{ \begin{gathered} x = vt \hfill \\ 2y = gt_{}^{2} \hfill \\ \end{gathered} \right. $$

where

2 $$ v = 2{\uppi }nl $$

In the formula (1) and formula (2) : *v* is the initial horizontal velocity of the center of mass of the rice seed at the moment of throwing, m / s, and its direction is the same as the forward direction of the planter; *l* is the distance between the centroid of the rice seed and the seeding shaft at the moment of throwing, m, *n* is the rotation speed of the seed-metering device, r / min, *t* is the time of the rice seed to do the flat throwing motion, s ; *x*, *y* are the coordinate value of the center of mass of the rice seed after the horizontal throwing motion time, m ; *g* is the acceleration of gravity, g = 9.8 m/s^2^.

After the time *t*_*1*_, the trajectory of the rice seed and the cylindrical surface of the outer diameter of the internal impeller intersect at point *a*(*x*_1_, *y*_1_), as shown in Fig. 2a.

It can be seen from Fig. 2a that the critical condition for rice seeds to enter the internal impeller compartment is:

3 $$ r\sin (\delta { + }\theta ) \ge x_{1} + \frac{{a_{\max } }}{2} $$

where $$\theta = 360nt_{1}$$.

*r* is the internal impeller radius, m; *d* is the impeller installation offset angle, °; θ is the angle of the impeller blade turning in time *t*_1_, the above style is arranged as follows:

4 $$ r \ge \frac{{2x_{1} + a_{\max } }}{{2\sin (\delta { + }\theta )}} $$

In the formula (4), *x*_1_ and θ are related to the rotation speed of the seed-metering device, and *a*_max_ is the maximum length size of the rice seed. Therefore, when the seed-absorbed cylinder is determined, the minimum radius of the internal impeller is related to the size of the rice seed and the offset angle at a certain seeding speed.

In order to guide the rice seed to slide from the internal impeller to the external impeller, the internal impeller compartment is set as an oblique surface, as shown in Fig. 2b.

It can be seen from Fig. 2b

5 $$ \left\{ \begin{gathered} \frac{{r - r_{1} }}{{l_{2} }} = {\text{tan}}\alpha \hfill \\ l_{1} + \frac{d}{2} \le l_{2} \hfill \\ \end{gathered} \right. $$

In the formula (5): *r*_1_ is the radius of the bottom circle of the internal impeller blade, m; *l*_2_ is the axial width of internal impeller blade, m; α is the angle between the slope and its rotation center line; *l*_1_ is the distance between the central axis of the middle suction hole and the outer end face of the seed-absorbed cylinder, m; *d* is the diameter of the socket entrance, m; the dimensions of *l*_1_ and *d* are 16 mm and 17 mm, respectively.

In order to ensure that the rice seed can smoothly enter the external impeller compartment along the inclined surface of the internal impeller, the angle α should not be less than the sliding friction angle γ between the rice seed and the internal impeller material. When the rice seed moves from the internal impeller to the external impeller, the minimum spacing of the blades should be able to pass through a single grain of rice seed, that is

6 $$ \frac{{2\pi r_{1} }}{k} - b \ge a_{\max } $$

In the formula (6): *a*_max_ is the maximum length of rice, m. *k* is the number of internal impeller compartments. *b* is the width of the blades, m. In this paper, take *b*=1.5 mm. Comprehensive formula (5) and formula (6):

7 $$ r - \left( {l_{1} + \frac{d}{2}} \right)tan\gamma \ge r_{1} \ge \frac{{k\left( {b + a_{{{\text{max}}}} } \right)}}{2\pi } $$

In order to shorten the movement time of rice seeds in the Internal impeller compartment, the bottom circle radius *r*_1_ of the internal impeller blade should be reduced as much as possible, so *r*_1_ = 31 mm is designed in this paper.

#### Cover plate

In order to prevent the rice seeds in the seed-filling area from entering the internal impeller compartment, it is necessary to set up an inner wall between the seed population and the internal impeller. The structure of the inner panel is shown in Fig. 1.

When the seed-metering device works, the falling rice seeds in the clearing area have a certain initial velocity along the rotation direction of the socket ring. The upper vertical plate can prevent the falling rice seeds in the clearing area from entering the internal impeller. With the rotating motion of the internal impeller, when there are more rice seeds in the same hole under the condition of replay, some rice seeds may stop on the inclined surface of the internal impeller and do not enter the external impeller; when the internal impeller blades are inclined downward, the remaining rice seeds in the internal impeller compartment flow back to the filling area along the internal impeller blades to ensure that each internal impeller compartment enters the seed-grouping arc section again in a seedless state.

The outer plate of the cover plate is equipped with an external impeller, which forms a sealed rotating compartment with the external impeller to prevent the outflow of rice seeds entering the external impeller compartment and other external impurities such as stones from entering the external impeller.

There is a seed outlet on the outer plate, and its position directly affects the consistency of rice seed throwing in the external impeller compartment. The angle of the seed outlet position φ is the angle between the plane where the seed outlet and the seeding shaft are located and the lead hammer surface is also the throwing angle.

**Figure 3:**
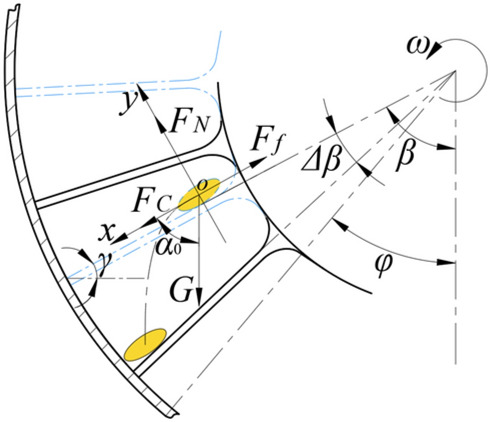
Schematic diagram of seed casting angle. Note: *G* is the gravity of rice seeds, *F*_*C*_ is the centrifugal inertia force of rice seeds, *F*_*N*_ is the support force of leaves to rice seeds, and *F*_*f*_ is the friction force.

When the angle between the external impeller blade and the horizontal plane is not less than the sliding friction angle γ of the rice seed, the rice seed begins to move downward along the blade, and the angle between the blade and the lead hammer surface is set to β. As shown in Fig. 3 below.

Through the pre-test observation of impeller seed guide, it can be seen that the movement of multi-grain rice seeds in the same hole on the separator is mostly sliding. Therefore, a simplified mechanical model for single-grain rice seeds is established in this paper, as shown in Fig. 3: and the very few collisions between rice seeds are ignored. When the sliding time of the rice is *t*_2_, the rotation angle of the impeller is *Δ*β, then:

8 $$ \Delta \beta_{} = \omega t_{2} $$

In the formula: ω is the rotation angular velocity of the impeller, rad/s.

The force equation of rice seed is as follows (10):

9 $$ \left\{ \begin{gathered} G{\text{cos}}\alpha_{0} + F_{C} - F_{f} = m\sigma \hfill \\ G = mg \hfill \\ F_{C} = m\omega^{2} l_{4} \hfill \\ F_{f} = \mu F_{N} \hfill \\ F_{N} = G{\text{sin}}\alpha_{0} \hfill \\ \end{gathered} \right. $$

In the formula: *m* is the quality of rice seeds, *g*; μ is the sliding friction coefficient between rice seed and impeller material, μ = tan γ; α_0_ is the angle between gravity and blade; *l*_4_ is the distance between the center of mass of rice seeds and the seeding shaft, m; σ is the acceleration of rice seed movement, m/s^2^.

Combining Eqs. (8) and (9), we get:

10 $$ \sigma = g\sqrt {\upmu ^{2} + 1} {\text{sin}}\left( {\omega t_{2} } \right) + \omega^{2} l_{4} $$

The rice seeds have various postures after gathering between the blades of the External impeller. In order to simplify the analysis, this paper refers to the equivalent radius *R* of the equal volume sphere *V*, and its calculation formula is Eq. (11):

11 $$ \left\{ \begin{gathered} 2R = \sqrt[3]{{\frac{6V}{\pi }}} \hfill \\ V = 0.523ahc \hfill \\ \end{gathered} \right. $$

The blade movement distance *L* of rice seeds satisfies Eq. (12):

12 $$ L = l_{4} - r_{2} - R $$

According to the analysis of Eq. (11), when the impeller rotates at a constant speed, the movement of rice seeds is variable acceleration. Integrate the movement process of rice seeds

13 $$ L = \iint\limits_{\phantom{0}} \sigma dt^{2} \;(t\epsilon \left( {0,t_{2} } \right]) $$

The equation of the relative leaf movement distance L of rice seeds can be obtained by combining equations (10), (12) and (13) as equation (14).

14 $$ L = \frac{{2g\omega t_{2} \sqrt {\upmu ^{2} + 1} + \omega^{4} t_{2}^{2} \left( {R + r_{2} } \right) - 2g\sqrt {\upmu ^{2} + 1} {\text{sin}}\left( {\omega t_{2} } \right)}}{{\left( {2 - \omega^{2} t_{2}^{2} } \right)\omega^{2} }} $$

It can be seen from the formula that when the impeller diameter and material are determined, the movement distance of the rice seed on the blade is only related to the rotation speed and time. When the seeding angle is large, a multi-grain rice seed may not have enough time to run out of the leaves, resulting in its collection before seeding. When the seeding angle is small, some of the rice seeds in the same hole are retained on the outer plate of the cover plate, which makes the agglomeration effect of the rice seeds in the same hole before seeding poor. Therefore, it is necessary to clarify the appropriate seeding angle to improve the agglomeration effect of rice seeds in the same hole before seeding, improve the consistency of seeding, and then improve the uniformity of seeding and hole spacing.

### Seed guide performance test

#### Seed-grouping performance and Seeding uniformity test

In order to comprehensively investigate the performance of one-hole multi-grain seeding and fixed-distance seeding of impeller seeding guide parts, and to clarify the better structural parameters of seeding guide parts, the seed-grouping performance test, seeding uniformity test and seeding uniformity comparison test of seeding guide parts were carried out in turn.

Whether a multi-grain rice seed can accurately fall into the same compartment of the inner impeller after being thrown out from the same socket is the first important index to detect the performance of the seed guiding parts. Therefore, the same-hole rate is taken as the evaluation index of the seed-grouping performance of the introduction parts, and the calculation formula is as follows:

15 $$ Y = \frac{w}{W} \times 100\% $$

In the formula (15):

*w* is the number of holes where multiple rice seeds fall into the same compartment of the internal impeller in the same socket; *Y* is the same-hole rate, %;

*W* is the sample size of each group in the seed-grouping performance test, the specific value is 250. The test repeat 3 times and take the average as result.

**Figure 4:**
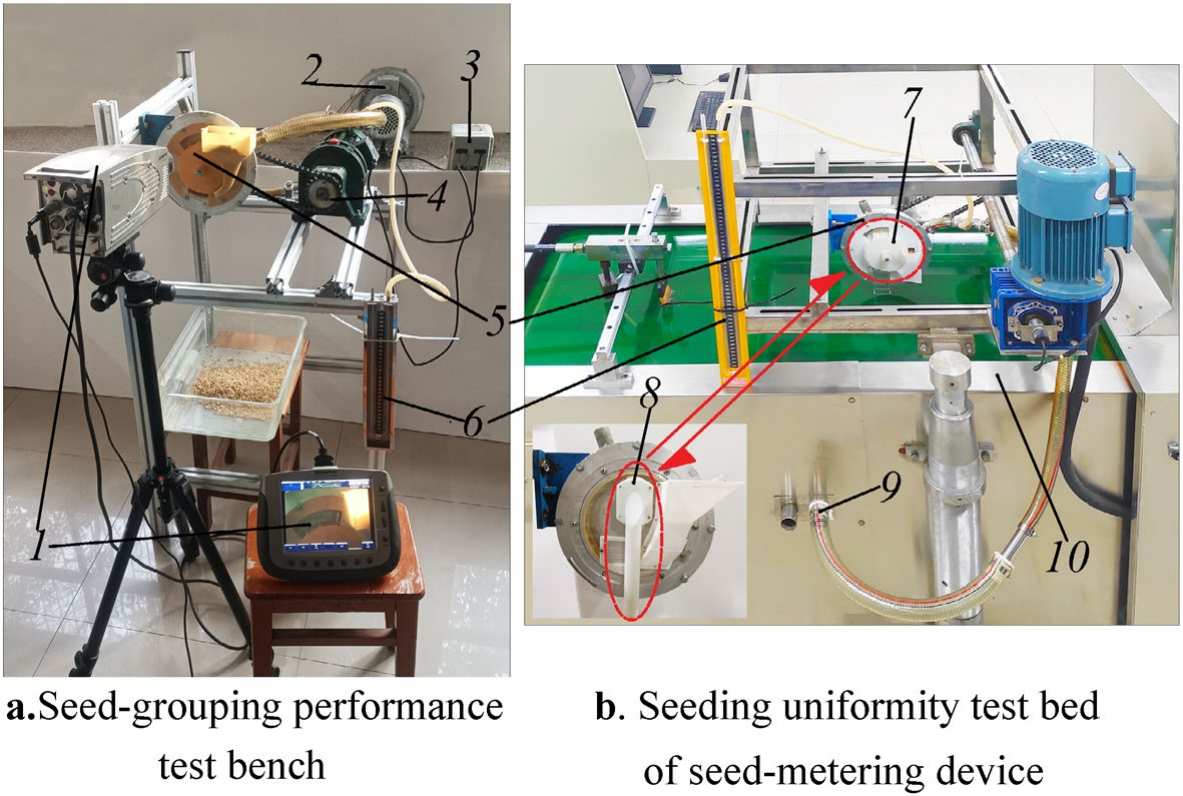
Two seed guide performance test. Note: 1. High-speed camera 2. Negative pressure fan 3. Frequency converter 4. Decelerating AC motor 5. Seed- metering device 6. U- type pressure gauge 7. Impeller 8. Seed guide tube 9. Negative pressure source 10. JPS-12 type performance test rig of seed-metering device.

The test device is shown in Fig. 4a. The negative pressure fan provides the required suction negative pressure for the seed-metering device. The U-type pressure gauge is used to detect the working negative pressure of the seed-metering device. The decelerating AC motor driven by the frequency converter provides rotating power for the seed-metering device. The high-speed camera is used to record the rice seed dropped into the same compartment of the impeller in each eye sock decelerating AC motor et.

In order to clarify the appropriate radius size parameters of the internal impeller and the blade offset angle (the angle between the internal impeller blade and the central axis of the suction hole), this paper first carried out a single factor test of the blade offset angle. After clarifying the appropriate parameter range of the blade offset angle, the two-factor full-factor test of the blade offset angle and the internal impeller radius was carried out. According to the angle of the internal impeller blade compartment is 25.71°, the blade offset angle is set to 5°, 10°, 15°, 20°, 25°, a total of five levels, and the internal impeller radius is set to 48, 52, 56 mm, a total of three levels.

Because Chuangliangyou 4418 is longer than Zhongnong 2008 and Yangguang 800, it is the most difficult to fall into the same compartment of the internal impeller. Therefore, this experiment only needs to select the Chuangliangyou 4418 rice seed with the longest grain shape as the seeding object, and clarify the influence of different internal impeller radius and blade offset angle on the same-hole rate.

The seeding position of the seed guiding component directly affects the consistency of the multi-grain rice seeds in one hole, which in turn affects the hole-forming and uniformity of the seed implantation. Therefore, the suitable throwing angle of the seed guiding component is very important to the hole forming and hole spacing uniformity of the seed-metering device. The test device is shown in Fig. 4b.

The experiment was carried out according to the test method of working quality of film-laying dibbler^18^; the average value of hole diameter (the straight line distance between the farthest rice seeds in a hole), the qualified rate of hole diameter (hole diameter ≤ 50 mm is qualified)^19,20^, the average value of hole distance (the center distance between two adjacent holes) and the coefficient of variation of hole distance were used as evaluation indexes. Continuous statistics of 250 holes of rice seeds discharged by the seed-metering device on the oil belt during stable seeding, each group of experiments was repeated three times, and the average value was taken. The calculation formula of test evaluation index is as follows:

16 $$ \left\{ \begin{gathered} D_{1} = \frac{{\sum {\left( {d_{1} } \right)_{i} } }}{{W_{1} }} \hfill \\ H = \frac{{w_{1} }}{{W_{1} }} \times 100\% \hfill \\ D_{2} = \frac{{\sum {\left( {d_{2} } \right)_{i} } }}{{W_{2} }} \hfill \\ C_{1} = \sqrt {\frac{{\sum {\left( {d_{2} - D_{2} } \right)^{2} } }}{{\left( {W_{2} - 1} \right)D_{2}^{2} }}} \times 100\% \hfill \\ \end{gathered} \right. $$

In the formula (16): *d*_1_ is the hole diameter, mm; *W*_1_ is the total number of hole diameter samples (250); *D*_1_ is the average hole diameter, mm; *w*_1_ is the qualified number of hole diameter; *H* is the qualified rate of hole diameter, %; *d*_2_ is the hole distance, mm; *W*_2_ is the total number of hole distance samples (250); *D*_2_ is the average value of hole spacing, mm; *C*_1_ is the coefficient of variation of hole spacing, %.

#### Comparative test of seed guide performance

On the basis of clarifying the optimal seed-grouping structure parameters and suitable seeding angle of the internal impeller, taking different varieties of rice seeds as the seeding object, the seeding performance comparison test under different seeding methods was carried out on the JPS-12 computer vision seed-metering device seeding performance test bench, and the excellent hole-forming and seeding-guiding effect of the impeller seeding device and its adaptability to the variety were clarified.

### Ethical approval

We confirm that all plant experiments and field studies involved in this study meet local and national regulatory requirements. We have obtained all the necessary permits to collect plant materials and have followed guidelines for conducting experiments. All procedures were conducted in accordance with the guidelines. There no human participants were involed during the study.

## Result and discussion

### Single factor test of blade offset angle

To determine the suitable range of blade offset angles, an inner impeller with a radius of 52 mm was selected^21^. The seed grouping rate *Y* was measured at different offset angles, specifically 5°, 10°, 15°, 20°, and 25°. The experimental results show that when the radius of the inner impeller is constant, the seed grouping increases first and then decreases with the increase of the blade offset angle. When the blade offset angle is in the range of 10°–20°, the rice seeds in the same hole are more likely to fall into the same internal impeller chamber, and the seed rate is higher, and the highest is 92.73%, all above 85%. The smaller or larger impeller offset angle, the lower the seed-grouping rate. It is indicated that the suitable blade offset angle should be between 10° and 20°.

### Two-factor full-factor test

In order to investigate the influence of different radius of the internal impeller and the interaction with different blade offset angles on the seed-grouping rate, to explore the optimal parameter combination of the internal impeller radius and the blade offset angle, and to improve the seed-grouping rate of the seed guide components, a two-factor full-factor test with different internal impeller radius and different blade offset angles was carried out. The effect of inclination angle, forward speed and rotational speed on miss-seeding rate was explored by Guo et al. through the F-test and analysis of variance^22^. The method was used to. This analysis method is aimed at high-speed inclined corn metering device. According to the single factor test results of blade offset angle, three levels of blade offset angle of 10°, 15° and 20° are taken. The test scheme and results are shown in the Table 1.

**Table 1:** Full factorial test scheme and results (Supplementary file 2).

Serial number	various factors and levels		*Y* (%)
	*A*: Radius of internal impeller (mm)	B: Offset angle of inner impeller (°)	
1	48	10	85.86
2	48	15	86.53
3	48	20	80.75
4	52	10	87.54
5	52	15	89.72
6	52	20	93.68
7	56	10	90.59
8	56	15	96.41
9	56	20	98.00

The regression analysis of the test results in the Table 1 was carried out by using the Design-Expert13 software, and the regression model equation of the vaccination rate was obtained as follows:

17 $$ Y = 0.214289 + 0.030317A - 0.06077B + 0.001564AB - 0.00039A^{2} - 0.00059B^{2} $$

In the formula: A and B are the radius of the inner impeller and the blade offset angle respectively. The results of variance analysis are shown in the Table 2.

**Table 2:** Factor test analysis of variance results.

Source	Sum of Squares	df	Mean Square	*F*-value	*p* value	
Model	0.0225	5	0.0045	9.43	0.0470	Significant
A	0.0169	1	0.0169	35.38	0.0095	
B	0.0012	1	0.0012	2.48	0.2132	
AB	0.0039	1	0.0039	8.20	0.0644	
${A}^{2}$	0.0001	1	0.0001	0.1625	0.7138	
${B}^{2}$	0.0004	1	0.0004	0.9204	0.4081	
Residual	0.0014	3	0.0005			
Cor Total	0.0240	8				

It can be seen from the table that the regression model is extremely significant, indicating that the regression model has high fitting accuracy with the actual results and can be used for the analysis and prediction of the seed-grouping performance. In addition, the radius of the internal impeller has a significant effect on the vaccination rate, while the blade offset angle has no significant effect on the vaccination rate within the range of test parameters, and the interaction between the two has no significant effect on the vaccination rate.

### Parameter optimization

In order to clarify the optimal parameter combination of inner impeller radius and blade offset angle, a single-objective optimization model (formula) of vaccination rate was established. The Design-Expert13 software was used to optimize the target within the range of test parameters. The inner impeller radius was 55.92 mm, the blade offset angle was 19.43°, and the vaccination rate was up to 98.71%.

In order to verify the accuracy of the solution results of the optimization model, an internal impeller with a radius of 56 mm was used, and the offset angle of the blade was set to 19.45°, and the seed-grouping rate *Y* was measured. Through the Eq. (17), the theoretical seed-grouping rate *Y* can be calculated be 98.63%. The results are showing in the Table 3, and the average vaccination rate of the three verification tests was 98.60%, and there was only a relative error with the solution value of the optimization model, indicating that the optimization model was reliable. The best parameter combination was the inner impeller radius of 56 mm and the blade offset angle of 19.5°.

**Table 3:** Single factor experiment of seeding angle.

Seeding angle	Average hole diameter (mm)	Qualified rate of hole diameter (%)	Average hole distance (mm)	Coefficient of variation of hole distance (%)
26°	27.0	90.4	200.4	12.0
31°	25.3	91.7	201.2	11.4
36°	19.7	96.1	201.5	10.1
41°	21.0	96.5	200.5	12.3

### Single factor test of seeding angle

The experimental design and results are shown in Table 3.

From the analysis of Table 3, it can be seen that with the increase of the seeding angle, the average value of the hole distance fluctuates on the theoretical hole distance of 200 mm, and the average value of the hole diameter and the coefficient of variation of the hole distance show a trend of decreasing first and then increasing. When the seeding angle is 36°, the minimum value is obtained, and the qualified rate of the hole diameter shows an increasing trend. When the seeding angle increases from 36° to 41°, the growth of the qualified rate of the hole diameter tends to be gentle. This is because when the seeding angle is small, the convergence distance of rice seeds in the outer plate is longer. Due to the influence of factors such as the shape and size of rice seeds and random collision, the time of arrival of multiple rice seeds in one hole to the seed outlet is quite different. With the increase of seeding angle, the convergence distance of rice seeds gradually became shorter, the consistency of seeding was improved, and the hole formation and hole spacing uniformity of seeding were significantly improved. However, when the seeding angle was too large, the multi-grain rice seeds in one hole failed to achieve effective convergence, and the uniformity of seeding and hole spacing decreased again. Li et al. also carried out the bench test, and used the omission rate, repeated rate, and pass rate as performance evaluation indexes^23^. Qualified rate of hole diameter and Coefficient of variation of hole distance as performance evaluation indexes are more suitable for the seed metering device mentioned in this paper.

### Comparative test

**Figure 5:**
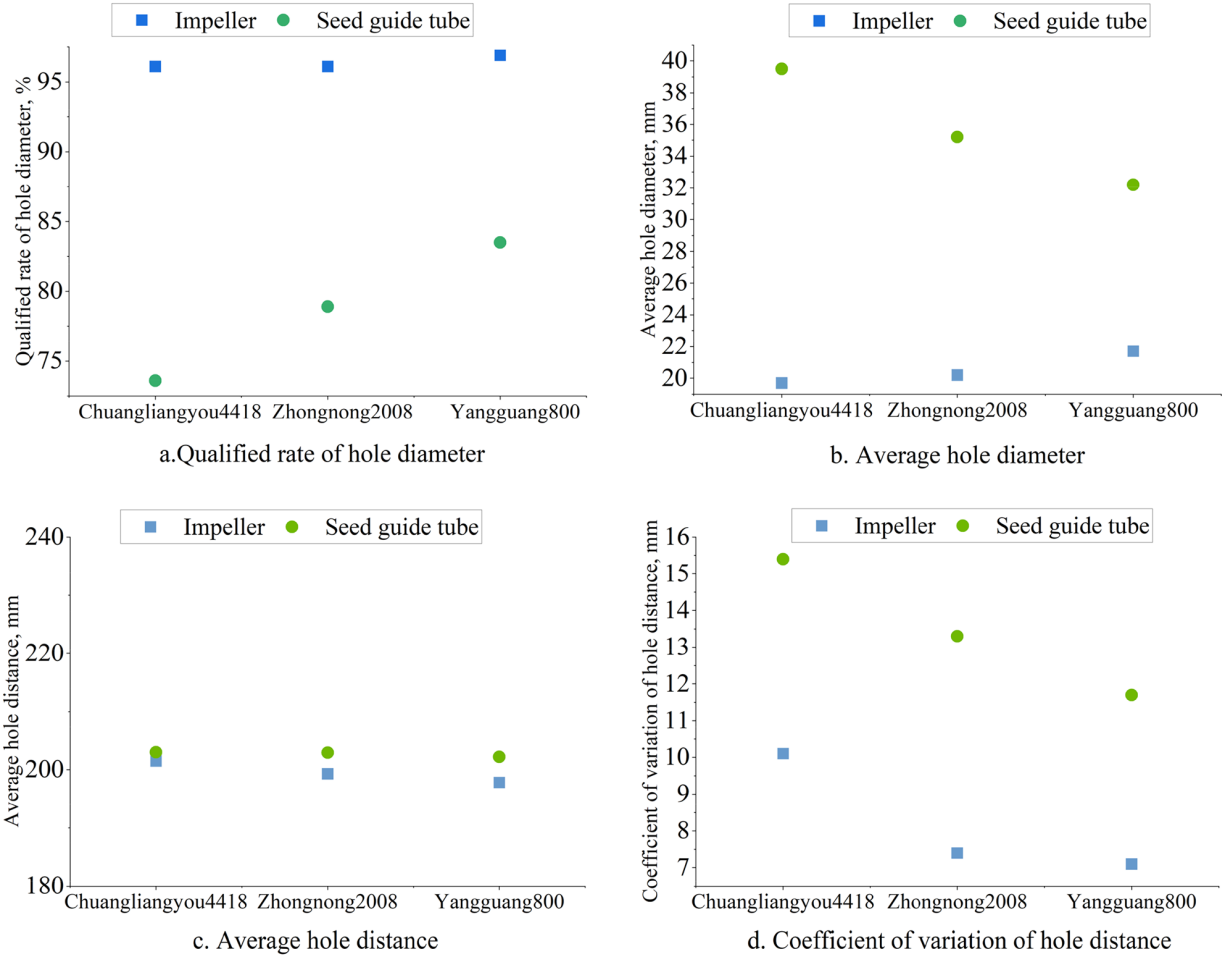
The hole seeding performance using different seed guide device.

The results of the comparative test of the performance using different seed guide device are shown in Fig.  5.

From Fig. 5, Taking the same kind of rice seeds as the seeding object, the average hole distance (Fig. 5c) of the seed-metering device is extremely close to the theoretical hole spacing of 200 mm under different seed guiding methods. However, the average hole diameter (Fig. 5b) and the coefficient of variation of hole distance (Fig. 5d) under the impeller seed guiding method are low, and the qualified rate of hole diameter (Fig. 5a) is high. The reason may be that the multi-grain rice seeds in the same hole have a large degree of freedom of movement in the seed guiding tube. Under the influence of mechanical vibration and random collision between rice seeds and rice seeds, rice seeds and pipe wall, it is difficult to maintain good seed setting and hole spacing uniformity. The impeller seed guiding device always maintains the synchronization of the movement of multi-grain rice seeds in a hole and the uniformity of the hole spacing by using the blade compartment during the seed guiding process. At the same time, the impeller seed guiding reduces the height of the seed, which is also conducive to the uniformity of the seeding. Therefore, compared with the seed guide tube, the impeller seed guide has good cavitation and hole spacing uniformity.

Under the impeller seeding method, the average hole diameter and the qualified rate of the hole diameter of the three varieties of rice seeds were similar. Compared with the other two rice varieties, the coefficient of variation of the hole spacing of Chuangliangyou 4418 was relatively large. The main reason was that the rice seed of Chuangliangyou 4418 was more slender than other rice varieties, which made the consistency of the seed input poor, and the seed input trajectory was more seriously affected by its own flip. It can be seen that no matter what kind of guidance method, the shorter and rounder the rice seed, the more conducive to the hole formation and hole spacing uniformity of the seeding.

Although the uniform effect of the impeller seed guiding device is also adversely affected by the shape of the slender rice seed, the coefficient of variation of the hole distance is basically less than 10%, and the average hole diameter is not higher than 21.7 mm, and the qualified rate of the hole diameter is not less than 96.1%. It can better meet the requirements of fixed-distance hole seeding for rice seeds of different shapes and sizes.

Taking the same kind of rice seeds as the seeding object, comparing the two seeding methods, it can be seen that the average hole diameter and the coefficient of variation of the hole spacing are lower under the impeller seeding method, and the qualified rate of the hole diameter is higher. The reason may be that the multi-grain rice seeds in the same hole have a large degree of freedom of motion in the seeding tube. Under the influence of mechanical vibration and the collision between rice seeds and rice seeds, rice seeds and pipe walls, it is difficult to maintain good seeding and hole formation and hole spacing uniformity. In the process of seeding, the impeller seeding device always maintains the synchronization of the movement of multi-grain rice seeds in one hole and the hole formation of seeding, and the uniformity of hole spacing. At the same time, the impeller seeding reduces the seeding height and is also beneficial to the uniformity of seeding.

Therefore, compared with the seed guide tube, the impeller seed guide has good cavitation and hole spacing uniformity.

## Conclusion

This paper presents an impeller seed guiding device for fixed-distance hole-forming and seed guiding in rice cultivation. The device utilizes interval hole-dividing seed guiding, reduces seeding height, and achieves good uniformity in hole-forming and spacing. The study includes theoretical analysis of key component parameters and sequential performance tests. The main conclusions are as follows.

1. The radius of the internal impeller has a significant effect on the seed-grouping rate of the seed guide device, while the blade wheel offset angle has no significant effect on the seed-grouping rate within the test parameter range of 10°–20°, and the interaction between the two has no significant effect on the seed-grouping rate. The best parameter combination of the seed guide device is the inner impeller radius of 56 mm and the offset angle of 19.45°. At this time, the seed-grouping rate can reach 98.6%.
2. The suitable seeding angle of the seeding device is 36°. At this time, the evaluation indexes of seeding uniformity are relatively better. The average hole diameter, the qualified rate of learning diameter and the coefficient of variation of hole distance are 19.7 mm, 96.1% and 10.1%, respectively. The average hole distance is 201.5 mm, which is very close to the theoretical hole distance of 200 mm.
3. Compared with the traditional seed guide tube, the impeller seed guide has good hole formation and hole spacing uniformity, and has good adaptability to different sizes of rice seeds, but medium-length and short-round rice seeds have good hole spacing uniformity.

Many factors affecting the performance of the seed-metering device need to be further studied and analyzed. For example, the seed-grouping rate has two sides. According to the experimental observation, for the qualified seed suction condition, some of the rice seeds in the same hole fail to enter the inner impeller compartment, thus becoming the missed sowing state. However, for the re-inhalation condition, to a certain extent, it can reduce the re-seeding rate and improve the qualified rate of sowing. From the overall performance of the impeller seed guide device, it can be seen that the coefficient of variation of the hole spacing of the impeller seed guide is small, the seeding uniformity is good, and it has a good application prospect. The seed metering device developed in this paper has not been tested in the field, for different water content or pre-germinated seeds have not been tested. In addition, the influence of rotational speed on the metering device has not been explored. In the subsequent research process, the performance of the metering device will be tested for the above two problems, and the relevant parameters will be fine-tuned. After more experiments and the adjustment of the relevant parameters in the future, the performance of the seed metering device will be improved.

## Supplementary Information

Supplementary Information 1.

Supplementary Information 2.

Supplementary Information 3.

Supplementary Information 4.

Supplementary Information 5.

Supplementary Information 6.

## Data Availability

Data is provided within the manuscript or supplementary information files.
