# Supplementary material for: Design and experiment of impeller seed guide device for rice internal suction hole direct seeding device
Source: Sci Rep. 2024 Jun 10;14:13300. doi: 10.1038/s41598-024-64002-x (PMC11639696; doi:10.1038/s41598-024-64002-x)
Supplement: Supplementary file 5 — Supplementary Information 5. [file 41598_2024_64002_MOESM5_ESM.docx]

# **Supplementary information**

## Single factor test of blade offset angle

To determine the suitable range of blade offset angles, an inner impeller with a radius of 52 mm was selected. The seed grouping rate Y was measured at different offset angles, specifically 5°, 10°, 15°, 20°, and 25°. The experimental results show that when the radius of the inner impeller is constant, the seed grouping increases first and then decreases with the increase of the blade offset angle. When the blade offset angle is in the range of 10 ° ~ 20 °, the rice seeds in the same hole are more likely to fall into the same internal impeller chamber, and the seed rate is higher, and the highest is 92.73 %, all above 85 %. The smaller or larger impeller offset angle, the lower the seed-grouping rate. It is indicated that the suitable blade offset angle should be between 10 ° and 20 °.

## Two-factor full-factor test

In order to investigate the influence of different radius of the internal impeller and the interaction with different blade offset angles on the seed-grouping rate, to explore the optimal parameter combination of the internal impeller radius and the blade offset angle, and to improve the seed-grouping rate of the seed guide components, a two-factor full-factor test with different internal impeller radius and different blade offset angles was carried out. According to the single factor test results of blade offset angle, three levels of blade offset angle of 10 °, 15 ° and 20 ° are taken. The test scheme and results are shown in the Table 1.

| **serial number** | **various factors and levels** | | ***Y*** (**%)** |
| --- | --- | --- | --- |
|  | ***A*: Radius of Internal impeller** (mm) | **B： Offset angle of inner impeller** (°) |  |
| 1 | 48 | 10 | 85.86% |
| 2 | 48 | 15 | 86.53% |
| 3 | 48 | 20 | 80.75% |
| 4 | 52 | 10 | 87.54% |
| 5 | 52 | 15 | 89.72% |
| 6 | 52 | 20 | 93.68% |
| 7 | 56 | 10 | 90.59% |
| 8 | 56 | 15 | 96.41% |
| 9 | 56 | 20 | 98.00% |

**Table 1**. Full factorial test scheme and results

The regression analysis of the test results in the Table 1 was carried out by using the Design-Expert software, and the regression model equation of the vaccination rate was obtained as follows:

$Y=0.214289+0.030317A-0.06077B+0.001564AB-0.00039A^{2}-0.00059B^{2}$ (18)

In the formula: A and B are the radius of the inner impeller and the blade offset angle respectively. The results of variance analysis are shown in the Table 2.

| **Source** | **Sum of Squares** | **df** | **Mean Square** | ***F*-value** | ***p*-value** |  |
| --- | --- | --- | --- | --- | --- | --- |
| **Model** | 0.0225 | 5 | 0.0045 | 9.43 | 0.0470 | significant |
| **A** | 0.0169 | 1 | 0.0169 | 35.38 | 0.0095 |  |
| **B** | 0.0012 | 1 | 0.0012 | 2.48 | 0.2132 |  |
| **AB** | 0.0039 | 1 | 0.0039 | 8.20 | 0.0644 |  |
| $\boldsymbol{A}^{\boldsymbol{2}}$ | 0.0001 | 1 | 0.0001 | 0.1625 | 0.7138 |  |
| $\boldsymbol{B}^{\boldsymbol{2}}$ | 0.0004 | 1 | 0.0004 | 0.9204 | 0.4081 |  |
| **Residual** | 0.0014 | 3 | 0.0005 |  |  |  |
| **Cor Total** | 0.0240 | 8 |  |  |  |  |

**Table 2.** Factor test analysis of variance results

It can be seen from the table that the regression model is extremely significant, indicating that the regression model has high fitting accuracy with the actual results and can be used for the analysis and prediction of the seed-grouping performance. In addition, the radius of the internal impeller has a significant effect on the vaccination rate, while the blade offset angle has no significant effect on the vaccination rate within the range of test parameters, and the interaction between the two has no significant effect on the vaccination rate.

### Parameter optimization

In order to clarify the optimal parameter combination of inner impeller radius and blade offset angle, a single-objective optimization model (formula) of vaccination rate was established. The Design-Expert software was used to optimize the target within the range of test parameters. The inner impeller radius was 55.92 mm, the blade offset angle was 19.43 °, and the vaccination rate was up to 98.71%.

In order to verify the accuracy of the solution results of the optimization model, an internal impeller with a radius of 56 mm was used, and the offset angle of the blade was set to 19.45 °, and the seed-grouping rate *Y* was measured. Through the Eq. (18), the theoretical seed-grouping rate *Y* can be calculated be 98.63%. The results are showing in the Table 3, and the average vaccination rate of the three verification tests was 98.60%, and there was only a relative error with the solution value of the optimization model, indicating that the optimization model was reliable. The best parameter combination was the inner impeller radius of 56 mm and the blade offset angle of 19.5 °.

## Single factor test of seeding angle

The experimental design and results are shown in Table 3.

| **seeding angle** | **Average hole diameter** (mm) | **Qualified rate of hole diameter** (%) | **Average hole distance** (mm) | **Coefficient of variation of hole distance** (%) |
| --- | --- | --- | --- | --- |
| 26 ° | 27.0 | 90.4 | 200.4 | 12.0 |
| 31 ° | 25.3 | 91.7 | 201.2 | 11.4 |
| 36 ° | 19.7 | 96.1 | 201.5 | 10.1 |
| 41 ° | 21.0 | 96.5 | 200.5 | 12.3 |

**Table 3.** Single factor experiment of seeding angle

From the analysis of Table 3, it can be seen that with the increase of the seeding angle, the average value of the hole distance fluctuates on the theoretical hole distance of 200 mm, and the average value of the hole diameter and the coefficient of variation of the hole distance show a trend of decreasing first and then increasing. When the seeding angle is 36 °, the minimum value is obtained, and the qualified rate of the hole diameter shows an increasing trend. When the seeding angle increases from 36 ° to 41 °, the growth of the qualified rate of the hole diameter tends to be gentle. This is because when the seeding angle is small, the convergence distance of rice seeds in the outer plate is longer. Due to the influence of factors such as the shape and size of rice seeds and random collision, the time of arrival of multiple rice seeds in one hole to the seed outlet is quite different. With the increase of seeding angle, the convergence distance of rice seeds gradually became shorter, the consistency of seeding was improved, and the hole formation and hole spacing uniformity of seeding were significantly improved. However, when the seeding angle was too large, the multi-grain rice seeds in one hole failed to achieve effective convergence, and the uniformity of seeding and hole spacing decreased again.

## Comparative test

The results of the comparative test of the performance using different seed guide device are shown in Fig.5.


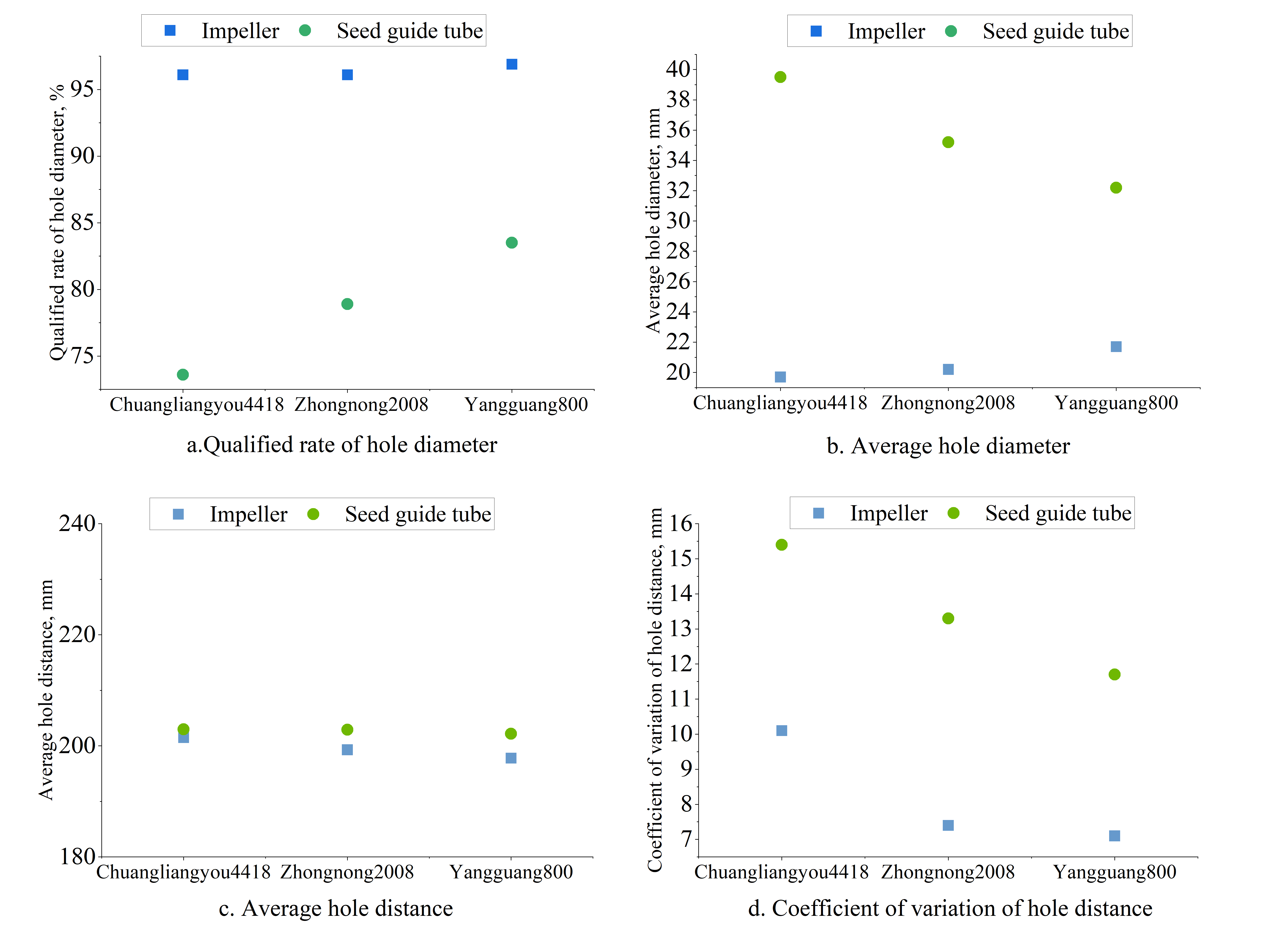


**Figture.5.** The hole seeding performance using different seed guide device

From Fig.5, Taking the same kind of rice seeds as the seeding object, the average hole distance (Fig.5c) of the seed-metering device is extremely close to the theoretical hole spacing of 200 mm under different seed guiding methods. However, the average hole diameter (Fig.5b) and the coefficient of variation of hole distance(Fig.5d) under the impeller seed guiding method are low, and the qualified rate of hole diameter(Fig.5a) is high. The reason may be that the multi-grain rice seeds in the same hole have a large degree of freedom of movement in the seed guiding tube. Under the influence of mechanical vibration and random collision between rice seeds and rice seeds, rice seeds and pipe wall, it is difficult to maintain good seed setting and hole spacing uniformity. The impeller seed guiding device always maintains the synchronization of the movement of multi-grain rice seeds in a hole and the uniformity of the hole spacing by using the blade compartment during the seed guiding process. At the same time, the impeller seed guiding reduces the height of the seed, which is also conducive to the uniformity of the seeding. Therefore, compared with the seed guide tube, the impeller seed guide has good cavitation and hole spacing uniformity.

Under the impeller seeding method, the average hole diameter and the qualified rate of the hole diameter of the three varieties of rice seeds were similar. Compared with the other two rice varieties, the coefficient of variation of the hole spacing of Chuangliangyou 4418 was relatively large. The main reason was that the rice seed of Chuangliangyou 4418 was more slender than other rice varieties, which made the consistency of the seed input poor, and the seed input trajectory was more seriously affected by its own flip. It can be seen that no matter what kind of guidance method, the shorter and rounder the rice seed, the more conducive to the hole formation and hole spacing uniformity of the seeding.

Although the uniform effect of the impeller seed guiding device is also adversely affected by the shape of the slender rice seed, the coefficient of variation of the hole distance is basically less than 10%, and the average hole diameter is not higher than 21.7 mm, and the qualified rate of the hole diameter is not less than 96.1%. It can better meet the requirements of fixed-distance hole seeding for rice seeds of different shapes and sizes.

Taking the same kind of rice seeds as the seeding object, comparing the two seeding methods, it can be seen that the average hole diameter and the coefficient of variation of the hole spacing are lower under the impeller seeding method, and the qualified rate of the hole diameter is higher. The reason may be that the multi-grain rice seeds in the same hole have a large degree of freedom of motion in the seeding tube. Under the influence of mechanical vibration and the collision between rice seeds and rice seeds, rice seeds and pipe walls, it is difficult to maintain good seeding and hole formation and hole spacing uniformity. In the process of seeding, the impeller seeding device always maintains the synchronization of the movement of multi-grain rice seeds in one hole and the hole formation of seeding, and the uniformity of hole spacing. At the same time, the impeller seeding reduces the seeding height and is also beneficial to the uniformity of seeding.

Therefore, compared with the seed guide tube, the impeller seed guide has good cavitation and hole spacing uniformity.
